# Supplementary material for: Discovery of a cofactor-independent inhibitor of Mycobacterium tuberculosis InhA
Source: Life Sci Alliance. 2018 Jun 1;1(3):e201800025. doi: 10.26508/lsa.201800025 (PMC6238539; doi:10.26508/lsa.201800025)
Supplement: Supplementary file 1 [file LSA-2018-00025_TableS1.pdf]

**Supplemental Table S1: Data collection and refinement statistics**			
	**InhA+AN2918**	**InhA+AN3438**	**InhA+AN12855**
**Protein**	InhA (R5645)	InhA	InhA
**Compound**	AN2918.01 / NAD+	AN3438.02 / NADH	AN12855 (EBSI 41882)
**Beamline**	APS-21-ID-F	APS-21-ID-G	APS 21 ID-F
**Space Group**	SG=5; C2	SG=20; C2221	$I4_122$
**Unit Cell**	a=125.35Å	a=81.66Å	*a*=94.08Å
	b=92.68Å	b=100.94Å	*b*=94.08Å
	c=101.93Å	c=377.82Å	*c*=184.43Å
	β=105.539		
**Solvent content**	50.41%	45.70%	
**Vm**	2.48 Å3/Da	2.26 Å3/Da	
**Resolution**	49.09-2.50Å (2.565-2.500)	48.76-2.55Å (2.62-2.55)	50-2.65Å (2.72-2.65)
**I/σ**	22.82 (4.63)	11.72 (3.14)	26.8 (3.85)
**Completeness**	99.9% (100.1%)	99.4% (99.7%)	99.6% (100%)
**Rmerge**	0.059 (0.515)	0.121 (0.506)	0.063 (0.502)
**Multiplicity**	6.8 (7.5)	5.3 (5.1)	9.6 (9.8)
**Reflections**	38978 (2872)	51162 (3740)	12,408 (896)
**Mosaicity**	0.21	0.30	0.20
**R**	0.191 (0.263)	0.235 (0.301)	0.172 (0.245)
**Rfree**	0.221 (0.327)	0.273 (0.342)	0.198 (0.243)
**Ramachandran**			
**Favored**	94.69%	96.23%	248 (95.8%)
**Allowed**	100.0%	100.0%	9 (3.5%)
Outliers			2 (0.8%), none per molprobity
**Molprobity Score**	1.18 (100th percentile)	1.65 (99th percentile)	1.31 (100th percentile)
**PDB File**	5VRM	5VRM	5VRL
