# Supplementary material for: Discovery of a cofactor-independent inhibitor of Mycobacterium tuberculosis InhA
Source: Life Sci Alliance. 2018 Jun 1;1(3):e201800025. doi: 10.26508/lsa.201800025 (PMC6238539; doi:10.26508/lsa.201800025)
Supplement: Supplementary file 2 [file LSA-2018-00025_Preparation_of_compounds.pdf]

Preparation of compound AN6534

Synthetic Scheme:

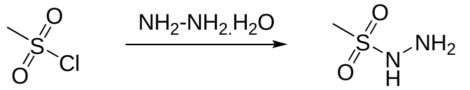

I **II**

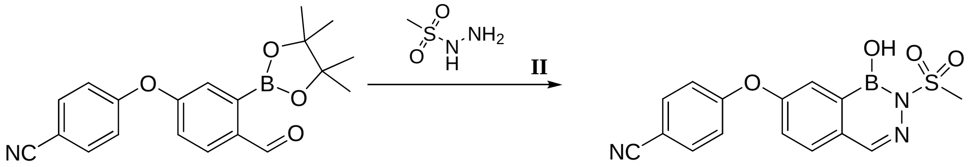

III **AN6534**

To a solution of compound **I** (15 g, 0.131 mol) in THF (200 mL) was added hydrazine hydrate (8.24 mL, 0.144 mol) in THF (100 mL) drop-wise at 0°C. The mixture was stirred at RT overnight. The mixture was filtered and washed with ACN. The filtrate was concentrated to give compound **II** (5 g, yield 35; **^{1}H NMR** (DMSO-d_6 400 MHz) δ 2.87 (s, 3H), 4.35 (br, 2H), 7.72 (br, 1H).

To a solution of compound **III** (Xia et al., 2011) (200 mg, 0.573 mmol) in EtOH (10 mL) was added compound **II** (63 mg, 0.573 mmol). The mixture was stirred at 50°C for 12 hrs. The mixture was cooled, the resulting solid was filtered and washed with EtOH to give **AN6534** (96 mg, yield 49%); ^{1}H NMR (DMSO-d_6 400 MHz) δ 8.26 (s, 1H) 7.95-7.82 (m, 4H) 7.60-7.57 (m, 1 H) 7.25-7.23 (d, J = 8.4 Hz, 2H) 3.36 (s, 1H).

Preparation of compound AN12541

Synthetic Scheme:

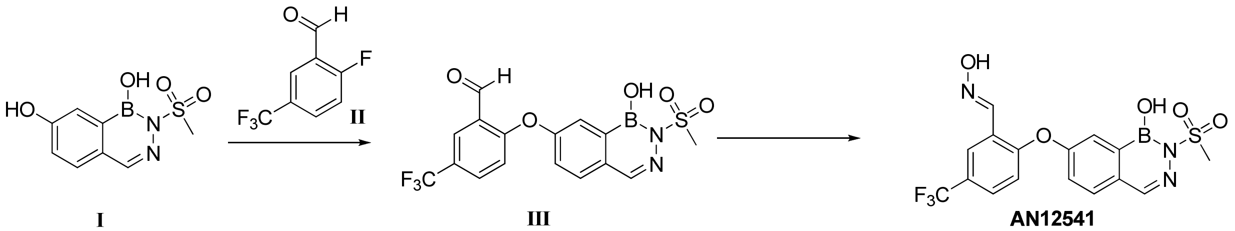

To a mixture of **compound I** (3.50 g, 14.58 mmol, 1.00 Eq) and **compound II** (5.60 g, 29.16 mmol, 2.00 equiv) in DMF (40 mL) , was added K_2CO_3 (4.03 g, 29.16 mmol, 2.00 equiv) in one portion at room temperature. The mixture was stirred at 80 °C. After4 h. the reaction mixture was cooled to room temperature. Water was added and the the mixture was extracted with ethyl acetate (3x100mL). The combined organic phase was washed with saturated brine (2x200 mL), dried with anhydrous Na_2SO_4, filtered and concentrated in vacuo to afford compound **3** (5.00 g, 12.13 mmol, 83% yield)

To a mixture of compound **III** (2 g, 4.86 mmol, 1.00 equiv) in EtOH (40 mL), was added hydroxyl amine hydrochloride (506.58 mg, 7.30 mmol, 1.50 Eq), KOAc (1.38 g, 15.08 mmol, 2.00 equiv) in one portion at r.t. The mixture was stirred at 50 °C After 2 h. the EtOH was removed in vacuo, the mixture was cooled to r.t.Water was added and the mixture was extracted with ethyl acetate (3x100mL). The combined organic phase was washed with brine (2x200 mL), dried with anhydrous Na_2SO_4, filtered and concentrated in vacuo, The crude material was purified via pre-HPLC to afford **AN12541** (860 mg, 53% yield); ^{1}H NMR (400 MHz DMSO-d_6) δ 11.77 (s, 1 H), 8.75 (br. s., 1 H), 8.26 (d, *J* = 16.1 Hz, 2 H, 8.12 (s, 1 H), 7.93 (d, *J* = 8.5 Hz, 1 H), 7.81 (d, *J* = 8.5 Hz, 1 H), 7.73 (d, *J* =2 .0 Hz, 1 H), 7.58 (dd, *J* = 8.5, 2.3 Hz, 1 H), 7.23 (d, *J* = 8.5 Hz, 1 H), 3.36 (s, 3 H).

Preparation of compound AN12855

Synthetic Scheme:

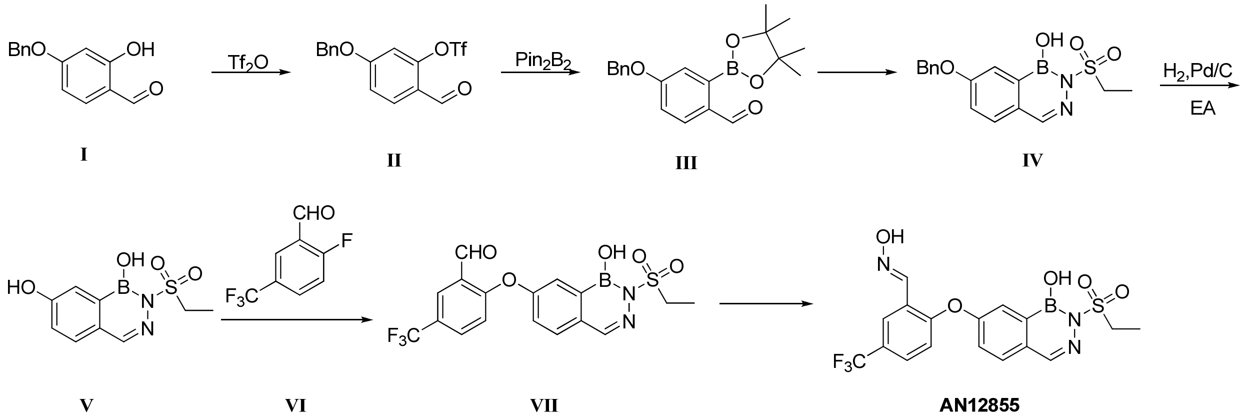

To a mixture of compound **I** (410.00 g, 1.80 mol, 1.00 eq), TEA (455.36 g, 4.50 mol, 2.50 equiv) and pyridine (28.48 g, 360.00 mmol, 0.2 eq) in DCM (1.5 L) was added Tf_2O (609.42 g, 2.16 mol, 1.20 Eq) dropwise at 0 °C. The mixture was warmed to 25 °C for 1 h. The mixture was poured into ice water and adjusted to pH 4-5, the aqueous phase was extracted with dichloromethane (2x1000 mL*2). The combined organic phase was washed with saturated brine (2x2000), dried with anhydrous Na_2SO_4, filtered and concentrated in vacuum, purified via column chromatography (EtOAc/petroleum ether = 5% ~ 10%) to afford compound **II** as light yellow oil (480.00 g, 1.33 mol, 74% yield).

A mixture of compound **II** (480.00 g, 1.33 mol, 1.00 eq), BPD (354.63 g, 1.4 mol, 1.05 Eq), KOAc (261.05 g, 2.66 mol, 2.00 eq) and Pd(dppf)Cl_2 (21.72 g, 26.60 mmol) in dioxane (1.5 L) was de-gassed and then heated to 80 °C. After 18 h under N_2 the mixture was filtered, concentrated in vacuo to give a residue**,** which was pre-purified via column chromatography to afford the compound **III** as white solid (350.00 g, 1.03 mol, 78% yield); ^{1}H NMR **(**$CDCl_3$, 400 MHz) δ 10.38 (s, 1H), 7.94 (d, *J* = 8.4 Hz, 1H), 7.34-7.47 (m, 6H), 7.10 (dd, *J* = 8.4, 2.4 Hz, 1H), 5.16 (s, 2H), 1.41 (s, 12H).

A mixture of compound **III** (40.00 g, 118.27 mmol, 1.00 eq) and ethanesulfonyl hydrazide (14.68 g, 118.27 mmol, 1.00 eq) in EtOH (100 mL) was stirred at 50°C for 18 h. HCl (15 ml, 3M) was added and the reaction mixture was stirred at 50°C for 2 h. The mixture was cooled to 20°C and the resulting solid was filtered and washed with petroleum ether to afford compound **IV** as a white solid (31.00 g, 90.07 mmol, 76% yield); ^{1}H NMR (DMSO-d_6, 400 MHz) δ 8.65 (s,

1H), 8.19 (s, 1H), 7.78-7.83 (m, 2H), 7.32-7.53 (m, 6H), 5.26 (s, 2H), 3.54 (q, *J* = 7.2 Hz, 2H), 1.19 (t, *J* = 7.2 Hz, 3H).

To a solution of compound **V** (32.00 g, 92.97 mmol, 1.00 Eq) in EtOAc (500 mL) was added Pd/C (10%, 15 g). The suspension was degassed under vacuum and purged with H_2 several times. The mixture was stirred under H_2 (50 psi) at 20°C for 3 h. TLC (petroleum ether:EtOAc = 1:2) indicated the starting material was completely consumed. The reaction mixture was filtered and the filtrate was concentrated to give compound **V** (23.00 g, 90.53 mmol, 97% yield) as a white solid; ^{1}H NMR (DMSO-d_6, 400 MHz) δ 10.36 (s, 1H), 8.32 (s, 1H), 8.12 (s, 1H), 7.70 (d, *J* = 8.4 Hz, 1H), 7.50 (d, J = 2.0 Hz, 1 H), 7.22 (dd, *J* = 8.4, 2.4 Hz, 1H), 3.54 (q, *J* = 7.2 Hz, 2H), 1.19 (t, *J* = 7.2 Hz, 3H).

To a mixture of compound **V** (1.00 g, 3.94 mmol, 1.00 eq) and compound **VI** (1.51 g, 7.88 mmol, 2.00 eq) in DMF (20 mL), was added K_2CO_3 (1.09 g, 7.88 mmol, 2.00 eq) in one portion at 20 °C. The mixture was warmed to 80 °C. After 4h the mixture was cooled to RT. Water was added and the mixture was extracted with EtOAc (3x100 mL). The combined organic phase was washed with saturated brine (2x200 mL), dried with anhydrous Na_2SO_4, filtered and concentrated in vacuo to afford compound **VII** (2.00 g, crude).

To a mixture of compound **VII** (1.50 g, 3.52 mmol, 1.00 eq) in EtOH (30 mL), was added hydroxylamine hydrochloride (366.91 mg, 5.28 mmol, 1.50 eq), KOAc (690.91mg, 7.04 mmol, 2.00 eq). The mixture was warmed to 50 °C. After 2h the EtOH was removed in vacuo. The mixture was extracted with EtOAc (3x100mL). The combined organic phase was washed with saturated brine (2x200 mL), dried with anhydrous Na_2SO_4, filtered and concentrated in vacuo, the residue was purified via prep-HPLC to afford compound **AN12855** (868.00 mg, 1.97 mmol, 56% yield) as a yellow solid; MS (ESI): mass calcd. For $C_{17}H_{15}BF_3N_3O_5S$ 441.08, m/z found 442 $[M+H]^+$; ^{1}H NMR (DMSO-d_6, 400 MHz) δ 11.78 (s, 1H), 8.80 (br. s., 1H), 8.27 (d, *J* = 12.4 Hz, 2H), 8.12 (br. s., 1H), 7.94 (d, *J* = 8.4 Hz, 1H), 7.81 (d, *J* = 7.2 Hz, 1H), 7.74 (d, *J* = 2.0 Hz, 1H), 7.56-7.63 (m, 1H), 7.24 (d, *J* = 8.4 Hz, 1H), 3.53 (q, *J* = 7.2 Hz, 2H), 1.19 (t, *J* = 7.2 Hz, 3H).

Preparation of compound AN12908

Synthetic Scheme:

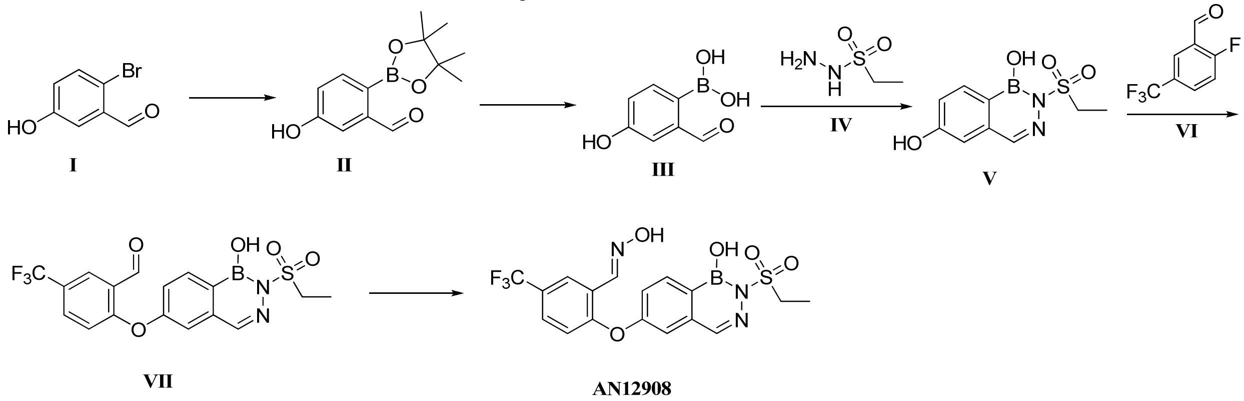

Compound **I** (10.00 g, 49.75 mmol, 1.00 Eq), Pin_2B_2 (13.27 g, 52.24 mmol, 1.05 Eq), KOAc (14.65 g, 149.25 mmol, 3.00 Eq) and $Pd(dppf)Cl_2.CH_2Cl_2$ (2.06 g, 2.49 mmol, 0.05 Eq) in dioxane (100 mL) was de-gassed and then heated to 80 °C. After 3 h the mixture was concentrated in vacuo to give a residue**,** which was purified via column chromatography to afford compound **II** (10.20 g, 41.12 mmol, 83% yield); ^{1}H NMR ($CDCl_3$ 400 MHz) δ 10.66 (s, 1 H) 7.87 (d, *J* = 8.3 Hz, 1 H) 7.50 (br. s., 1 H) 7.10 (d, *J* = 6.5 Hz, 1 H) 5.70 (s, 1 H) 1.38 (s, 12 H).

To a solution of compound **II** (10.20 g, 41.12 mmol, 1.00 Eq) in THF/H_2O (80/80 mL) was added $NaIO_4$ (43.98 g, 205.60 mmol, 5.00 Eq) and the mixture was stirred at r.t. After 3 h the reaction was filtered. The filtrate was extracted with EtOAc. The organic layer was washed with brine, dried over Na_2SO_4 and concentrated (5.90 g, 35.56 mmol, 87% yield).

The mixture of compound **III** (800.00 mg, 4.82 mmol, 1.00 Eq) and compound **IV** (598.58 mg, 4.82 mmol, 1.00 Eq) in EtOH (30 mL) was stirred at 50 °C overnight. The solvent was removed in vacuo to give compound **V** (1.3 g, crude).

To a mixture of compound **V** (800.00mg, 3.15mmol, 1.00 Eq) and compound **VI** (907.72 mg, 4.72 mmol, 1.50 Eq) in DMF (30 mL), was added K_2CO_3 (870.37 mg, 6.30 mmol, 2.00 Eq) in one portion at r.t. under N_2 and the reaction mixture was heated to 80 °C. After 2 hthe mixture was cooled to r.t. The residue was poured into water and the mixture was extracted with EtOAc. The organic phase was washed with saturated brine, dried with anhydrous Na_2SO_4, filtered and

concentrated in vacuo. The residue was purified via silica gel chromatography to afford compound **VII** (900.00 mg, crude).

To a mixture of compound **VII** (150.00 mg, 351.97 umol, 1.00 equiv) and $NH_2OH{\cdot}HCl$ (36.69 mg, 527.95 umol, 1.50 Eq) in EtOH (15 mL), was added KOAc (69.08 mg, 703.94 umol, 2.00 equiv) in one portion at r.t. under N_2. Then heated to 50 °C and stirred for 1 h. HPLC showed the reaction was completed. The solvent was removed. The residue was poured into water. The mixture was extracted with EtOAc. The organic phase was washed withbrine, dried with anhydrous Na_2SO_4, filtered and concentrated in vacuum. The residue was purified via prep-HPLC to afford **AN12908** (78.00 mg, 176.79 umol, 50% yield) as a white solid; ^1H NMR (400 MHz d_6-DMSO) δ 11.80 (s, 1 H) 8.82 (br. s., 1 H) 8.22 - 8.34 (m, 3 H) 8.13 (s, 1 H) 7.83 (d, *J* = 8.03 Hz, 1 H) 7.43 - 7.55 (m, 2 H) 7.29 (d, *J* = 9.03 Hz, 1 H) 3.56 (q, J=7.19 Hz, 2 H) 1.21 (t, *J* = 7.40 Hz, 3 H)

Sequencing of clinical isolates

DNA was extracted from the clinical isolates using the MasterPure Gram positive DNA purification kit (Lucigen) following heat inactivation at 90°C for 60 min. DNA was stored at 4°C. PCR was performed using the following primer pairs fabG1-inhA (TB-FabG1-MMF1, TCAATACACCCGCAGCCA and InhA-R1, GTGATACCCCACCGAAATGC), or katG (TB-KatG-MMF3, TCTATACCGGACTACGCC and TB-KatG-MMR4, TGGACCGTTTCGACAA). Reactions were performed with Platinum Taq DNA polymerase High Fidelity (Invitrogen) in the presence of 5% (v:v) DMSO and 1M betaine (pH 9) (Sigma). Regional sequence of the resultant amplicons was performed using nested primer sets. Mutations in *katG* were associated with isoniazid resistance for all three strains of interest. Low-level isoniazid resistance in TN5904 was associated with a previously described G944C mutation ($KatG_{S315T}$) previously shown to retain catalase-peroxidase activity in vitro (Ando AAC 2010). High level isoniazid resistance was associated with a frameshift mutation in *katG* (ΔG803), resulting in premature termination at

AA343 in strain M70. We repeatedly failed to amplify the *katG* region from strain M28, suggesting a partial or complete deletion or rearrangement of this region. Such deletions have been reported previously (Ando AAC 2010). No mutations in *inhA* or the *fabGI* promoter were observed for any of the strains.

Supplemental References

Xia Y, Cao K, Zhou Y, Alley MR, Rock F, Mohan M, Meewan M, Baker SJ, Lux S, Ding CZ, Jia G, Kully M, Plattner JJ (2011) Synthesis and SAR of novel benzoxaboroles as a new class of beta-lactamase inhibitors. Bioorg Med Chem Lett 21: 2533-6

Ando H, Kondo Y, Suetake T, Toyota E, Kato S, Mori T, Kirikae T (2010). Identification of *katG* mutations associated with high-level isoniazid resistance in *Mycobacterium tuberculosis*. Antomicrobial Agents and Chemotherapy. 54: 1793-1799
