# Supplementary material for: Discovery of a cofactor-independent inhibitor of Mycobacterium tuberculosis InhA
Source: Life Sci Alliance. 2018 Jun 1;1(3):e201800025. doi: 10.26508/lsa.201800025 (PMC6238539; doi:10.26508/lsa.201800025)
Supplement: Supplementary file 3 [file LSA-2018-00025_TableS2.pdf]

Supplemental Table 2: Pharmacokinetics of AN12855 in murine models.

**Mice strain**	**CD-1**			**C57Bl/6**						**BALB/c (infected)**	
**Route-Dose**	**IV-5 mg/kg**	**Oral-10 mg/kg**		**Oral-10 mg/kg**		**Oral-25 mg/kg**		**Oral-50 mg/kg**		**Oral-10 mg/kg**	**Oral-50 mg/kg**
**Number of Mice**	3	3		2		2		2		3	3
**Dosing Schedule**	Single dose	Single dose		QD-4 days		QD-4 days		QD-4 days		QD-3 wk	QD-3 wk
	Plasma	Plasma	Lung	Plasma	Lung	Plasma	Lung	Plasma	Lung	Plasma	Plasma
**Cmax (µg/mL)**	6.32	1.74	1.02	5.79	0.584	12.6	1.9	14.9	3.5	8.36	61.6
**Ratio Plasma Cmax/Dose**				0.58	-	0.5	-	0.3			
**Tmax (h)**		0.5	4	0.5	5	0.5	5	0.5	5	0.5	0.5
**AUClast (h*µg/mL)**	14.3	15.4	10.4	17.6	N/A	46.8	N/A	82.4	N/A	28.9	192
**AUC0-inf (h*µg/mL)**	14.4	15.5	-	19.7	-	59	-	109	-	29	192
**Ratio Plasma AUC0-last / Dose**				1.8	-	1.9	-	1.6			
**Terminal t1/2 (h)**	3.46	3.27	-	2.5	-	3.52	-	3.76	-	3.04	2.4
**CL (mL/h/kg)**	348										
**Vss (mL/kg)**	1270										
**MRTinf (h)**	3.64										
**Ratio (Lung / Plasma) @ 5 hr**				-	0.39	-	0.48	-	0.36		
**Ratio (Lung / Plasma) @ 8 hr**				-	0.51	-	0.49	-	0.47		
**Bioavailability (%)**		53	NA								
