# Supplementary material for: Discovery of a cofactor-independent inhibitor of Mycobacterium tuberculosis InhA
Source: Life Sci Alliance. 2018 Jun 1;1(3):e201800025. doi: 10.26508/lsa.201800025 (PMC6238539; doi:10.26508/lsa.201800025)
Supplement: Supplementary file 4 [file LSA-2018-00025_TableS3.pdf]

Table S3 : Efficacy of AN12855 in an Acute (C57BL/6) model of infection					
			Mean ± SEM Log10 CFU (Number of mice)		
Organ	Treatment	Conc (mg/kg)	Days post infection 1	13	22
Lung	Untreated		2.5 ± 0.08 (3)	7.2 ± 0.04 (5)	9.1 ± 0.05 (5)
Lung	INH	25			5.5 ± 0.20 (5)*
Lung	AN12855	0.1			8.8 ± 0.08 (5)
Lung	AN12855	1			8.4 ± 0.20 (5)*
Lung	AN12855	10			6.8 ± 0.10 (5)*
Lung	AN12855	25			6.4 ± 0.06 (5)*
Lung	AN12855	50			5.4 ± 0.23 (4)[A]*
Spleen	Untreated			5.4 ± 0.14 (5)	7.4 ± 0.12 (5)
Spleen	INH	25			4.1 ± 0.07 (5)*
Spleen	AN12855	0.1			7.0 ± 0.12 (5)
Spleen	AN12855	1			7.1 ± 0.18 (5)
Spleen	AN12855	10			5.4 ± 0.12 (5)*
Spleen	AN12855	25			4.2 ± 0.12 (5)*
Spleen	AN12855	50			3.9 ± 0.18 (5)*

*: P value <0.05 compared to untreated controls
A: Data only included for 4 of the 5 mice in this group due to plate contamination
