# Supplementary material for: Discovery of a cofactor-independent inhibitor of Mycobacterium tuberculosis InhA
Source: Life Sci Alliance. 2018 Jun 1;1(3):e201800025. doi: 10.26508/lsa.201800025 (PMC6238539; doi:10.26508/lsa.201800025)
Supplement: Supplementary file 5 [file LSA-2018-00025_TableS4.pdf]

Table S4: Efficacy of AN12855 in an Acute (C57BL/6) model of infection with dose escalation					
			Mean ± SEM Log10 CFU (Number of mice)		
Organ	Treatment	Conc (mg/kg)	Days post infection 1	13	22
Lung	Untreated		2.3 ± 1.09 (3)	6.6 ± 0.11 (5)	8.1 ± 0.07 (5)
Lung	INH	25			4.6 ± 0.06 (5)*
Lung	AN12855	50			5.8 ± 0.05 (5)*
Lung	AN12855	100			5.0 ± 0.13 (5)*
Lung	AN12855	200			5.6 ± 0.06 (5)*
Spleen	Untreated			3.9 ± 0.44 (5)	7.1 ± 0.18 (5)
Spleen	INH	25			2.4 ± 0.15 (5)*
Lung	AN12855	50			2.5 ± 0.24 (5)*
Lung	AN12855	100			2.5 ± 0.05 (5)*
Lung	AN12855	200			3.0 ± 0.10 (5)*
*: P value <0.05 compared to untreated controls					
