# Supplementary material for: Discovery of a cofactor-independent inhibitor of Mycobacterium tuberculosis InhA
Source: Life Sci Alliance. 2018 Jun 1;1(3):e201800025. doi: 10.26508/lsa.201800025 (PMC6238539; doi:10.26508/lsa.201800025)
Supplement: Supplementary file 6 [file LSA-2018-00025_TableS5.pdf]

Table S5 : Efficacy of AN12855 in an Chronic (BALB/c) model of infection							
			Mean ± SEM Log10 CFU (Number of mice)				
Organ	Treatment	Conc (mg/kg)	Days post infection-1	27	41	55	83
Lung	Untreated		2.0 ± 0.15 (3)	7.4 ± 0.07(5)	6.3 ± 0.13(5)	6.1 ± 0.27(5)	6.3 ± 0.11(5)
Lung	INH	25			5.7 ± 0.08(5)*	5.0 ± 0.07(5)*	3.7 ± 0.09(5)*
Lung	AN12855	5			6.2 ± 0.12(5)	6.3 ± 0.13(5)	6.4 ± 0.14(6)
Lung	AN12855	25			6.1 ± 0.10(5)	5.9 ± 0.03(5)	5.4 ± 0.07(6)*
Lung	AN12855	100			5.8 ± 0.08(5)*	5.3 ± 0.07(6)	4.5 ± 0.05(6)*
Spleen	Untreated			5.5 ± 0.09(5)	4.8 ± 0.16(5)	4.9 ± 0.17(5)	5.5 ± 0.05(5)
Spleen	INH	25			3.4 ± 0.04(5)*	2.4 ± 0.08(5)*	1.7 ± 0.00(3)[A]*
Spleen	AN12855	5			4.8 ± 0.14(5)	5.1 ± 0.09(5)	5.3 ± 0.11(5)
Spleen	AN12855	25			3.8 ± 0.10(5)*	4.0 ± 0.10(5)*	3.5 ± 0.19(5)
Spleen	AN12855	100			3.4 ± 0.10(5)*	2.9 ± 0.13(5)*	2.1 ± 0.10(5)[B]*

*: P value < 0.05
A: Data for two animals with CFU reported as the limit of detection, i.e. 50 CFU
B: Data for one animal with CFU reported as the limit of detection, i.e. 50 CFU
